# Associated factors and their individual contributions to caesarean delivery among married women in Bangladesh: analysis of Bangladesh demographic and health survey data

**DOI:** 10.1186/s12884-019-2588-9

**Published:** 2019-11-21

**Authors:** Farhana Hasan, Md. Mesbahul Alam, Md. Golam Hossain

**Affiliations:** Health Research Group, Department of Statistics, University of Rajshahi, Rajshahi, 6205 Bangladesh

**Keywords:** Caesarean delivery, BDHS-2014, Bangladeshi women, Step-wise regression, PAF

## Abstract

**Background:**

Caesarean section (CS) delivery has a significant effect on maternal and neonatal health especially in a developing country like Bangladesh. The aim of the study was to determine the risk factors and their individual contribution to CS delivery among Bangladeshi married women in reproductive age.

**Methods:**

The cross sectional secondary data was used in this study. Data was extracted from Bangladesh Demographic and Health Survey (BDHS), 2014 dataset. BDHS-2014 collected data from all over Bangladesh. Stepwise logistic regression analysis and population attributable fractions (PAF) were utilized in this study.

**Results:**

A total number of 4422 married Bangladeshi women having at least one child (age ≤ 5 years) were considered in this study. The prevalence of CS delivery among Bangladeshi women was 23.94%. The stepwise logistic regression model showed that location (division), type of residence, education of respondent and her husband, working status, age at first birth, number of children, wealth index and baby’s birth weight were most important predictors of CS delivery among Bangladeshi mothers. PAF demonstrated that overweight or obese women had highest contribution (23.36%) among the risk factors of CS delivery, followed by age at first birth (age >  20 years) (18.97%), highest wealth quintile (17.39%), higher education (15.93%), living in urban environment (14.39%), having lower number of ever born children (1–2 children) (13.58%), living in Dhaka division (12.11%), delivering large size of child at birth (11.13%) and housewife (6.55%).

**Conclusions:**

In the present study, we have identified the important risk factors and their individual contribution to CS delivery in Bangladesh. Consequently, these factors can be considered for reducing the rate of CS delivery in Bangladesh.

## Introduction

When vaginal delivery is not possible, caesarean section (CS) is an alternative mode of giving birth, which ensures the safety of mother and her child. CS delivery is a highly effective procedure with surgical intervention in obstetrical care for preventing birth complications of a mother. CS delivery causes various complications for mother and the birth could be traumatic for the baby with long term consequences. Some previous studies reported that deficiency of family bondage, disharmony and shock were associated with CS delivery [1, 2]. In 1985 WHO suggested that the optimal population range for CS delivery rates would be between 5 and 15% [3, 4], and WHO stated, in 2015 (BMG-2016) that for the crying need of CS delivery to a woman all possible effort should be provided without endeavoring any kind of specific rate [5]. During pregnancy maternal deaths are mainly caused by hemorrhage, unsafe abortion, hypertension, obstructed labour and infections. Though these types of complications are unpredictable, almost all could be prevented by ensuring institutional delivery services as timely management and treatment can make the difference between life and death [6, 7]. Lack of knowledge on CS delivery and misinformation about natural childbirth is important reasons for women to choose delivery by CS [8]. Also fear, anxiety and pain can play an important role for mothers to choose CS delivery [9]. Now a day in Bangladesh without any complications some women undergo CS delivery.

In Bangladesh, a remarkable number of CS deliveries were found among mothers who completed secondary education, and lived in urban areas and rich families [10]. Multiple aspects ranging from maternal, socio-demographic and institutional factors have been attributed to the increase of caesarean deliveries. It has been revealed by a couple of previous studies that upturns of mother ages increase the events of caesarean birth [11]. Another significant cause is marital age which causes the rising rate of caesarean birth in the developing countries [12]. The association of conveyed complications about delivery and some socio-demographic and pertinent characteristics of women having CS delivery have been explored by using a sample survey data [13]. Some other previous studies from different countries reported that various socio economic, demographic, physical facilities and community interrelated issues played a significant role in taking decisions to undergo institutional delivery [14–21].

To the best of our knowledge, most of the earlier studies determined the prevalence and associated factors of CS delivery. It is essential to indentify the most important predictors and their individual contribution to CS delivery. These types of risk factors can help to improve health policy for a particular population for reducing CS delivery where health and medically related reforms are being actively implemented.

The objective of this study was to investigate the associated factors and their individual contributions to CS delivery among Bangladeshi married women at the reproductive age.

## Methods

### Design and study population

In the present study, we used secondary data which was collected by Bangladesh Demographic and Health Survey (BDHS-2014). It was a nationally representative survey conducted from June 28, 2014 to November 9, 2014. The BDHS-2014 used a two-stage stratified cluster sampling method based on enumeration areas (EAs) and households. All information about sampling technique, survey design, survey instruments, measuring system, quality control, ethical approval and subject consent for the 2014 BDHS have been described elsewhere [10]. A total number of 4493 ever-married women of reproductive age (age 15–49) having at least one child (age ≤ 5 year) were available for the study sample. After removing outliers and cases with incomplete and missing data, the sample was reduced to 4422 for the analysis in the present study. The mean age of the women was 31.02 ± 9.22 years.

### Selected variables

The outcome variable in the present study was dichotomous variable, CS delivery, (i) No or (ii) Yes. This variable was measured by a question to participants, “Did you deliver your last baby by a caesarean?”

The primary choice of some explanatory variables for this study was based on other previous studies on the factors influencing CS delivery [11, 22, 23]. The socio-demographic factors such as living locations (division), type of residence, education, age at first marriage, age at first birth, number of children, current working status, religion, family member, child’s birth weight, wealth index and body mass index were used in this study. The socioeconomic, demographic and household information was included in our study that came from the questionnaires used in the BDHS-2014.

### Statistical analysis

In this study, an initial bivariate analysis (χ2-test) was performed to determine significant associations between **mode of birth** (caesarean vs. non-caesarean) and selected socio-demographic factors. These associated variables were considered as the independent variables for the stepwise logistic regression model, which was carried out in this study to find the most influential factors for CS delivery. Stepwise logistic regression analysis is a technique for selecting influential variables in multiple regression models [24]. Both methods, forward LR and backward LR, are used in stepwise logistic regression analysis to compute the final step by subsequently adding (forward LR) or taking away (backward LR) variables. Backward (LR) elimination may have the advantage that it is taking into consideration suppressor effects that might be lost in forward inclusion for logistic stepwise regression [25]. This method starts with the full equation and successively drops one variable at a time based on the Wald statistic value. When all the Wald statistic values are significant, the full set of variables is retained in the final step [25]. The backward (LR) elimination method in logistic stepwise regression was used in this study.

Finally, population attributable fraction (PAF) was utilized in the present study to calculate the contribution of each risk factor for CS delivery. PAF is used to calculate the individual contribution of influential risk factors, assuming that the exposure has a causal effect on the outcome. Reduction of proportion in average disease risk over a time period which is achieved by eradicating the exposure(s) of interest from the population is defined as the PAF. A PAF is calculated by using the odds ratios for interventional risk factors between two categories. After adjusting the other factors the formula is as follows:

PAF = pc (adjusted odds ratio (AOR) – 1)/adjusted odds ratio (AOR), where, pc is the prevalence of the factor among the cases [26].

Statistical significance was accepted at *p* < 0.05 and analyses were carried out using SPSS software (version IBM 20).

## Results

In the present study, a total number of 4422 ever-married Bangladeshi women aged between 15 and 49 having at least one child (age ≤ 5 years) were considered as a sample. Among them 1413 (31.95%) and 3009 (68.05%) came from rural and urban environment respectively. The prevalence of caesarean delivery among married women in Bangladesh was 23.94% (Table 1).

**Table 1:** Association between mode of birth and socioeconomic and demographic factors of Bangladeshi married women in reproductive age

Variables	Group	Overall sample, N (%)	Caesarean section, sN (%)	χ2-value	*p*-value
Division	Dhaka	780 (17.6)	257 (32.9)	139.607	*p* < 0.001
	Chittagong	849 (19.2)	171 (20.1)		
	Borishal	528 (11.9)	106 (20.1)		
	Khulna	524 (11.8)	186 (35.5)		
	Rajshahi	535 (12.1)	148 (27.7)		
	Rangpur	544 (12.3)	105 (19.3)		
	Sylhet	662 (15.0)	85 (12.8)		
Residence	Urban	1413 (32)	521 (36.9)	191.210	*p* < 0.001
	Rural	3009 (68)	537 (17.8)		
Education	No Education	596 (13.5)	45 (7.6)	507.362	*p* < 0.001
	Primary	1222 (27.6)	152 (12.4)		
	Secondary	2097 (47.4)	568 (27.1)		
	Higher	507 (11.5)	293 (57.8)		
Husband’s education	No Education	1013 (22.9)	92 (9.1)	525.610	*p* < 0.001
	Primary	1334 (30.2)	200 (15.0)		
	Secondary	1406 (31.8)	407 (28.9)		
	Higher	669 (15.1)	359 (53.7)		
Currently Working	No	3449 (78.0)	873 (25.3)	16.540	*p* < 0.001
	yes	973 (22.0)	185 (19.0)		
Age at first marriage	< 18 years	3736 (84.5)	761 (20.4)	167.352	*p* < 0.001
	≥18 years r	686 (15.5)	297 (43.3)		
Age at first birth	≤ 20 years	3596 (81.3)	706 (19.6)	194.920	*p* < 0.001
	> 20 years	826 (19.7)	352 (42.6)		
No. of children ever born	1–2 children	3117 (70.5)	882 (28.3)	110.847	*p* < 0.001
	> 2 children	1305 (29.5)	176 (13.5)		
Birth Weight	Normal	3836 (86.7)	882 (23.0)	13.848	*p* < 0.001
	Large	586 (13.3)	176 (30.0)		
Body mass index	Underweight (BMI ≤ 18.5 kg/m^2^)	1097 (24.8)	147 (13.4)	261.338	*p* < 0.001
	Normal weight (18.5 < BMI < 25 kg/m^2^)	2608 (59.0)	585 (22.4)		
	Overweight(25 ≤ BMI < 30 kg/m^2^)	627 (14.2)	274 (43.7)		
	Obese (BMI ≥ 30 kg/m^2^)	90 (2.0)	52 (57.8)		
Family member	≤ 4 persons	1379 (31.2)	365 (26.5)	7.118	0.004
	> 4 persons	3043 (68.8)	693 (22.8)		
Wealth Index	Poor	1774 (40.1)	155 (8.7)	517.331	*p* < 0.001
	Middle	854 (19.3)	169 (19.8)		
	Rich	1794 (40.6)	734 (40.9)		
Religion	Non-Muslim	355 (8.0)	100 (28.2)	3.818	0.031
	Muslim	4067 (92.0)	958 (23.6)		
Total		4422 (100)	1058 (23.94)		

It was noted that the highest number of CS delivery was found in Khulna (35.5%) division followed by Dhaka (32.9%), Rajshahi (27.7%), Chittagong and Barisal (20.1%), Rangpur (19.3%) and Sylhet (12.8%) divisions. χ^2^-test demonstrated that the association between living location (division) and mode of birth was statistically significant (*p* < 0.001). We observed that more urban women (36.9%) underwent CS delivery than rural (17.8%) women, and the association between these two factors was significant (*p* < 0.001). It was observed that CS delivery rate varied according to the level of education and the prevalence was higher among women with higher education (57.8%) along with their husbands’ education (53.7%). The association between level of education and CS delivery was significant (*p* < 0.001) for both women and their husbands. It was found that the prevalence of CS delivery was higher among women who were currently not working (25.3%) than those who were working (19.0%), and the association between these two factors was statistically significant (*p* < 0.001). The percentages of CS delivery were higher among women who got married after the age of 18 years (43.3%), and the association between these two factors was significant (*p* < 0.001). Higher number of CS deliveries were found among women who got their first baby after the age of 20 years (42.6%) than younger women, and the association between women’s age at first birth and their mode of birth was significant (*p* < 0.001). Women having 1 to 2 children had a higher prevalence of CS delivery (28.3%) than women with more than two children (13.5%), and the association between parity and mode of birth was significant (*p* < 0.001). The prevalence of CS delivery was higher among women who got a large-size baby at birth (30.0%), and the association between child’s birth size and the mode of birth was significant (*p* < 0.001). It was noted that the highest number of CS deliveries (57.8%) was found among obese women, and the association between women’s body size and their mode of birth was significant (*p* < 0.001). We observed that women living in a small family (member≤4) were more prone to get CS delivery than their large family counterparts, and the association between these two factors was significant (*p* < 0.001). CS delivery was more common among women living in rich households (40.9%) compared with others, and the association between wealth index and mode of birth was significant (*p* < 0.001). It was found that Non-Muslim women (28.2%) were more likely to undergo CS delivery than Muslim women, and the association between these factors two was significant (*p* < 0.05) (Table 1).

All the above significant factors were considered as independent variables in stepwise logistic regression model for getting most important risk factors for CS delivery. All independent variables were converted into two categories, (i) reference and (ii) non reference for calculating the contribution of each variable to CS delivery. Only important risk factors were considered for calculating their individual contribution to CS delivery by using PAF, and these factors came from the last step of stepwise logistic regression analysis.

### Stepwise logistic regression

Stepwise logistic regression model included all variables in the first step as predictors for CS delivery (Table 2). In the first step, we observed that the least significant variable based on the Wald statistic was the age at first marriage and the corresponding change in −2LR was also insignificant (*p* > 0.05); thus this variable was excluded from the model. It was observed in the second step that religion was the least significant variable and the change in −2LR was insignificant (*p* > 0.05); therefore, this variable was excluded from the model. In the third step, it was noted that family members was the least significant variable and the corresponding change in −2LR was insignificant (*p* > 0.05); thus this variable was excluded from the model. Finally, we observed that the variables namely living location (division), type of residence, education, and husband’s education, current working status, age at first birth, number of ever born children, wealth index, child’s birth weight and body mass index were included in step 4; all these variables were statistically significant (*p* < 0.01) for the change in -2LR; thus this step was the final step for this model (Table 2). Among our considered variables, these ten variables were the most important predictors for CS delivery of Bangladeshi married women during the reproductive age.

**Table 2:** Stepwise logistic regression (first and final steps) for socio-demographic factors and BMI influencing CS delivery

Step	Valuable (category)	β	Wald	*p*-value	AOR	95% CI for AOR		Change in -2LR
						lower	upper	
Step 1	Division ((Dhaka Vs Other than Dhaka (**Ref.**))	−0.46	21.54	0.001	1.59	1.31	1.93	21.12
	Residence ((Urban Vs Rural **(Ref**.))	−0.48	31.97	0.001	1.61	1.36	1.90	31.68
	Education ((Higher Vs Lower (**Ref.**))	0.66	40.81	0.001	1.94	1.58	2.38	41.92
	Husband’s education ((Higher Vs Lower (**Ref**.))	0.56	35.37	0.001	1.75	1.45	2.10	35.73
	Working status ((Housewife Vs Working outside (**Ref.**))	0.32	9.25	0.002	1.37	1.12	1.68	9.50
	Age at first birth ((More than 20 years Vs ≤20 years (**Ref.**))	0.43	11.67	0.001	1.54	1.20	1.97	11.52
	Age at first marriage ((≥18 years Vs < 18 years (**Ref**.))	0.15	1.30	0.254	1.16	0.90	1.50	1.13
	No. of family member ((≤4 persons Vs > 4 persons (**Ref.**))	0.13	2.36	0.124	0.88	0.74	1.04	2.35
	No. of ever born children ((1–2 Vs More than 2(**Ref**.))	−0.61	23.42	0.001	1.83	1.43	2.34	24.03
	Wealth index ((Rich Vs Poor or middle (**Ref.**))	0.84	60.08	0.001	2.31	1.87	2.85	63.58
	Child’s birth weight ((LargeVs Normal (**Ref.**))	0.47	18.02	0.001	1.60	1.29	1.99	17.52
	Body mass index ((Overweight or obese Vs Normal or under (**Ref.**))	0.71	52.31	0.001	2.03	1.67	2.46	51.46
	Religion ((Muslim Vs Non-Muslim (**Ref.**))	0.23	2.54	0.111	0.80	0.60	1.05	2.49
Final step (Step 4)	Division ((Dhaka Vs Other than Dhaka (**Ref.**))	−0.46	21.53	0.001	1.58	1.30	1.92	21.11
	Residence ((Urban Vs Rural (**Ref.**))	−0.49	34.74	0.001	1.64	1.39	1.93	34.43
	Education ((Higher Vs Lower (**Ref.**))	0.66	40.72	0.001	1.94	1.58	2.38	41.83
	Husband’s education ((Higher Vs Lower (**Ref.**))	0.56	36.23	0.001	1.76	1.46	2.11	36.61
	Working status (Housewife Vs Working outside (**Ref.**))	0.30	8.62	0.003	1.36	1.11	1.66	8.84
	Age at first birth ((More than 20 years Vs ≤20 years (**Ref.**))	0.52	26.88	0.001	1.68	1.38	2.04	26.41
	No. of ever born children ((1–2 Vs More than 2(**Ref.**))	−0.66	29.60	0.001	1.94	1.53	2.46	30.50
	Wealth index ((Rich Vs Poor or middle (Ref.))	0.82	58.00	0.001	2.26	1.83	2.79	61.34
	Child’s birth weight ((Large Vs Normal (**Ref.**))	0.46	17.53	0.001	1.59	1.28	1.97	17.05
	Body mass index ((Overweight or obese Normal or under (**Ref.**))	0.71	52.60	0.001	2.03	1.68	2.46	51.74

### Population attributable fractions (PAF)

The contribution of each important risk factor, living location (division), type of residence, education, and husband’s education, current working status, age at first birth, number of ever born children, wealth index, child’s birth weight and body mass index for CS delivery among Bangladeshi women were calculated using PAF after removing the effect of other factors. Among the important risk factors of CS delivery, overweight or obese women have the highest contribution (23.36%) with the burden (95% CI: 18.58–27.30) compared with normal or under weight, followed by age at first birth (age >  20 year) (18.97%; 95% CI: 15.46–21.82) compared with younger age at first birth, women living in a rich family (17.39%) with burden (95% CI: 12.85–21.95) compared with poor or middle family women. The next most significant factor was respondent’s husband (15.99%; 95% CI: 12.15–19.12) and her higher education level (15.93%; 95% CI: 11.70–19.46) compared with lower education, followed by urban women (14.39%; 95% CI:10.37–17.80) compared with rural women, number of children (1–2) (13.58%; 95% CI: 9.68–16.64) compared with more than two children, women living in Dhaka division (12.11%; 95% CI: 7.66–15.77) compared with those in other divisions, delivered large size child at birth (11.13%; 95% CI; 6.55–14.80) compared with normal birth weight and housewife (6.55%; 95% CI: 2.40–9.94) compared with women who were working outside of house (Table 3).

**Table 3:** PAF for risk factors of caesarean delivery among Bangladeshi women of reproductive age

Risk Factors	Comparison Group	PAF(%)	Range (95% CI)	
			Lower	Upper
Body mass index	Normal or under (**Ref.**)	23.36	18.58	27.3
	Overweight or obese			
Age at first birth	<=20 years (**Ref.**)	18.97	15.46	21.82
	More than 20 years			
Wealth Index	Poor or middle (**Ref.**)	17.39	12.85	21.95
	Rich			
Husband’s education	Lower (**Ref.**)	15.99	12.15	19.12
	Higher			
Education	Lower (**Ref**.)	15.93	11.7	19.46
	Higher			
Residence	Rural (**Ref.**)	14.39	10.37	17.80
	Urban			
No. of children	More than 2(**Ref**.)	13.58	9.68	16.64
	1–2			
Division	Other than Dhaka (**Ref.**)	12.11	7.66	15.77
	Dhaka			
Baby’s birth weight	Normal (**Ref.**)	11.13	6.55	14.8
	Large			
Working status	Working outside (**Ref.**)	6.55	2.4	9.94
	Housewife			

**Fig. 1:**
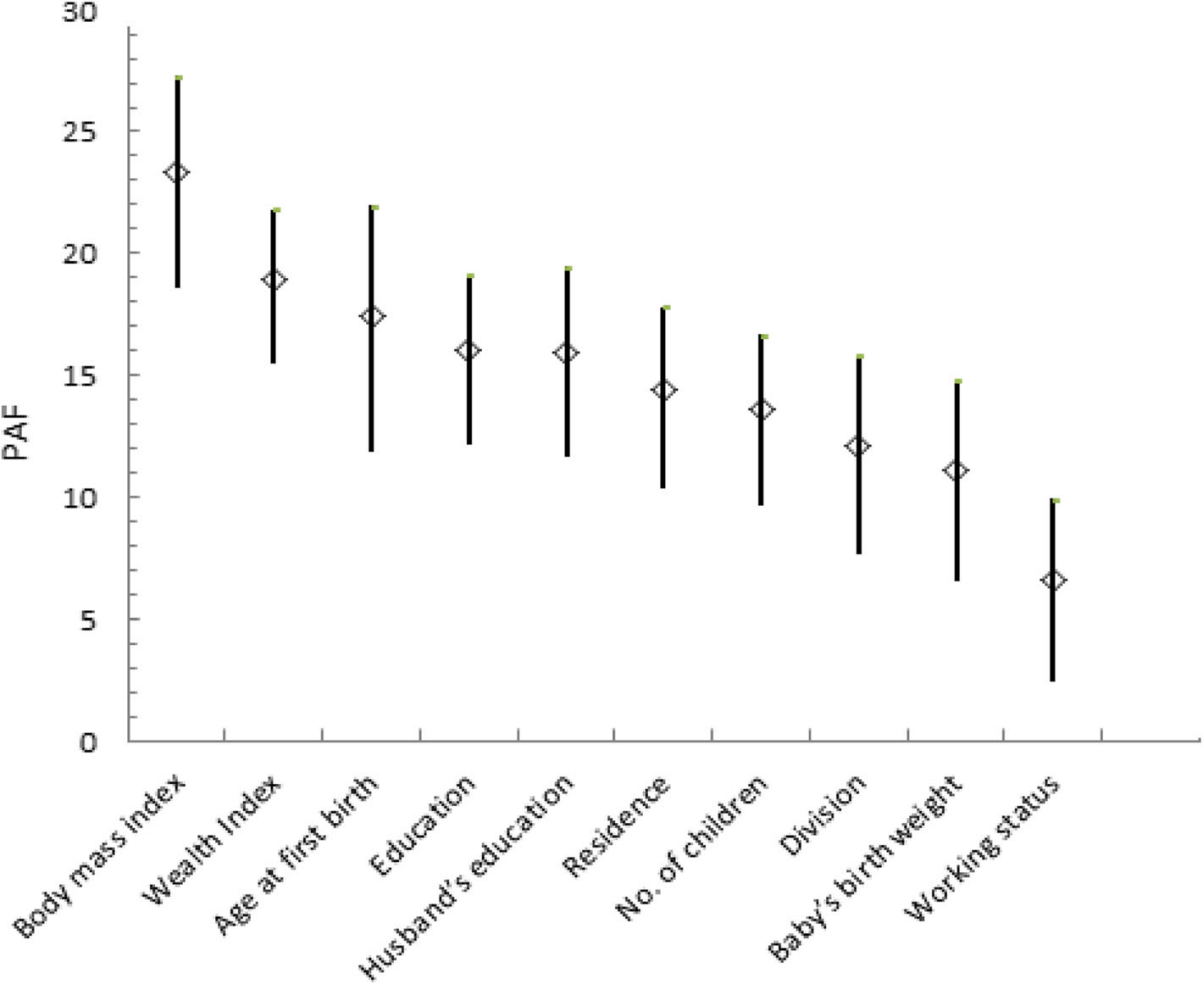
Individual contribution of risk factors on cesarean delivery among married women in Bangladesh

The PAF for each of the interventional significant risk factors are compared in the following figure. The figure showed that the exposure became more conservative, the PAF declined, and the confidence intervals narrowed (Fig. 1).

## Discussion

This study was aimed to determine the risk factors for CS delivery in Bangladesh by using nationally representative data. It was noted that the prevalence of CS delivery in Bangladesh was 23.94%, which was higher than the recommendation of WHO [5]. In a global survey, it was reported that the prevalence of CS delivery was highest in China (46.2%) [27]. Whereas the other countries ranged from 1.62% (Angola) to 42.0% (Paraguay) [28, 29]. In Latin America and the Caribbean region, the highest rate of CS delivery was (32%), while in the African region reported that the lowest rate of CS delivery was (7%) [30]. Variations of CS delivery rate in different regions worldwide might be due to cultural, educational and economic differences across areas.

The rate of CS delivery among Bangladeshi women has been increasing for the last 10 years [10, 31]. The household’s wealth index, female education level and medical facilities in Bangladesh have been increasing for the last two decades [10]. In Bangladesh now medical facilities are available even in small towns. Most of the women in urban or semi urban areas have ability to get proper nursing care during their pregnancy period. More than 74% women in urban areas are educated and they belong to middle-class and rich families, and have access to and ability to undergo CS delivery [10]. Now a day educated pregnant women want to avoid vaginal delivery in fear of labour pain and other consequences. Perhaps these are the most important reasons for the increased rate of CS delivery in Bangladesh. According to doctors, caesarean section is normally justified under certain complicated physical problems at delivery time. BDHS-2014 collected information on the reasons for which a doctor proposed CS delivery, and their findings showed that women underwent to CS delivery to avoid labor pain (3.2%), and in the cases of mal presentation (41.5%), premature baby (1.7%), cord prolapsed (2.6%), multiple births (0.2%), failure to progress in labor (21.1%), preeclampsia (2.9%), diabetes (0.5%), less pressure on baby’s brain (9.7%), convenience (5.8%) and other complications (38.6%) [10]. However, when there is no pregnancy or delivery related complications, even then some CS deliveries are performed.

Many studies on CS delivery had been done with different population, and they reported that some socio-demographic and anthropometric factors were related to CS delivery [10–13]. Most of these studies did not mention the important predictors for CS delivery and they did not estimate the contribution of individual variables. The important risk factors and their individual contribution to CS delivery among married women in a particular population would help to identify the vulnerable group for improving health policy and make awareness about the bad effect of CS delivery on maternal and neonatal health. Maybe this was the first time we attempted to determine the important risk factors and their individual contribution for CS delivery using two very powerful statistical techniques, stepwise logistic model and PAF. Stepwise logistic model provided that living location (division), type of residence, education of women and their husbands, current working status, age at first birth, number of children, household’s wealth index, child’s birth weight and categories of BMI were most important predictors for CS delivery among Bangladeshi married women at the reproductive age. PAF demonstrated that among the important risk factors for CS delivery, BMI had the highest contribution followed by age at first birth, household’s wealth index, respondent’s husband’s and her education level, type of residence, number of children, living location (division), child’s birth weight and current working status.

The findings of this study concerning parity portrayed that the odds of caesarean delivery decrease with an increase in number of children. A possible explanation is that after the birth of the first and the second child by caesarean section, subsequent deliveries are perceived to be of high risk, thus decreasing the likelihood of delivering subsequent babies. This reflects women’s perceptions regarding the efficacy of the procedure as a means to ensure the newborn baby’s survival and to avert the risks of birth complications or stillbirth. In this study, we found that comparatively old women were more likely to get CS delivery than younger women. This finding was supported by China study [2].

It was observed that urban women were twice as likely to undergo CS delivery as rural women. The possible explanation could be that educated and wealthy women have a greater confidence and capability to take actions regarding their own health [32, 33]. In Bangladesh, urban women are more educated and belong to higher wealth quintile household than rural women [10]. Surprisingly, working women were less likely to have caesarean delivery than housewives. These findings were supported by other studies; perhaps working women experience time constraints that reduce their opportunities for receiving antenatal care [34]. The rate of cesarean section with large birth weight was significantly higher than those of normal or low birth weight baby and this result was confirmed by other studies [35].

PAF provided that overweight is the strongest independent risk factor for CS delivery with the rate of 23.36% among Bangladeshi married women. Because in the pregnancy period if a woman becomes overweight or obese then risks of gestational diabetes and preeclampsia is increased, the fetus is also at risk for stillbirth and congenital anomalies [36]. In Bangladesh, urban women were twice as likely to be overweight or obese compared to rural women. Also the proportion of overweight or obese women had been increasing with increasing educational attainment and wealth index, and women with highest wealth quintile were more likely to be overweight or obese six times higher compared with women in the lowest wealth quintile [10]. If there was no overweight or obese mother among Bangladeshi married women, CS delivery would be reduced from this population. Other contributing risk factors for increasing the rate of CS delivery are that married women get babies after they are 20 years old, and women living in a rich family with higher education, living in an urban environment, having children not more than 2, living in Dhaka division, give birth to large weight baby. These factors can play an important role in reducing CS delivery rate in Bangladesh.

### Limitations of the study

The study was based on the most recent BDHS-2014 data with a nationally representative large sample size. Regardless of the above strengths, the study has several limitations. For example, besides the selected socio demographic factors which have been included in this analysis, a host of other programmatic factors, such as, accessibility, quality, and costs of delivery services, cultural factors, some social prejudice, lack of interest in taking care of child bearing women by other family members and women’s role in decision-making process are also likely to influence the delivery practices of women. However, due to lack of relevant data, in this study we were not able to examine the effects of such factors on the child-delivery practices. Also the availability of data did not permit us to examine all aspects of the delivery practices. Since BDHS-2014 did not collect the information from medical records of patients, we were not able to investigate the doctor’s medical grounds to operate for caesarean delivery.

## Conclusions

The prevalence of CS delivery among married women of reproductive age in Bangladesh is high, and it has increased during the last 10 years. The percentage of caesarean birth in Bangladesh is well above the World Health Organization recommendation of ideal rate for caesarean delivery. Women living in urban areas with higher education, living in rich households have significantly higher rates of caesarean delivery. Also, overweight and obesity, age at first birth and large baby at birth are important predictors for CS delivery.

These significant factors might be considered for reducing the rate of CS delivery among Bangladeshi mothers. This study suggests that health authorities of Bangladesh should arrange intervention programs for raising awareness about the bad effect of CS delivery especially among women who live in urban areas with high education and live in rich households.

## Data Availability

The datasets used in this study are freely available at http://dhsprogram.com/data/ dataset/ Bangladesh_Standard-DHS_2014.cfm?flag = 0.

## Abbreviations

AOR: Adjusted Odds Ratio
BDHS: Bangladesh Demographic and Health Survey
BMI: Body Mass Index
CS: Cesarean Section
EAs: Enumeration Areas
PAF: Population Attributable fractions
WHO: World Health Organization
